# Systematic review of incretin therapy during peri-operative and intensive care

**DOI:** 10.1186/s13054-018-2197-4

**Published:** 2018-11-14

**Authors:** Abraham H Hulst, Mark P Plummer, Markus W Hollmann, J Hans DeVries, Benedikt Preckel, Adam M Deane, Jeroen Hermanides

**Affiliations:** 1 Department of Anaesthesiology, Amsterdam UMC, University of Amsterdam, Meibergdreef 9, Postbus 22660, 1105 AZ Amsterdam, the Netherlands; 2 Intensive Care Unit, Royal Melbourne Hospital, 300 Grattan Street, Parkville, VIC 3050 Australia; 3 Department of Endocrinology, Amsterdam UMC, University of Amsterdam, Meibergdreef 9, Postbus 22660, 1105 AZ Amsterdam, the Netherlands

**Keywords:** DPP-IV inhibitors, GIP, GLP-1, Glucose control, Hyperglycaemia, Hypoglycaemia, Intensive care, Peri-operative care

## Abstract

**Background:**

Glucagon-like peptide 1 (GLP-1) and glucose-dependent insulinotropic peptide (GIP) are incretin hormones. By lowering blood glucose in a glucose-dependent manner, incretin-based therapies represent a novel and promising intervention to treat hyperglycaemia in hospital settings. We performed a systematic review of the literature for all current applications of incretin-based therapies in the peri-operative and critical care settings.

**Methods:**

We searched MEDLINE, the Cochrane Library, and Embase databases for all randomised controlled trials using exogenous GLP-1, GLP-1 receptor agonists, exogenous GIP and dipeptidyl peptidase IV inhibitors in the setting of adult peri-operative care or intensive care. We defined no comparator treatment. Outcomes of interest included blood glucose, frequency of hypoglycaemia and insulin administration.

**Results:**

Of the 1190 articles identified during the initial literature search, 38 fulfilled criteria for full-text review, and 19 single-centre studies were subsequently included in the qualitative review. Of the 18 studies reporting glycaemic control, improvement was reported in 15, defined as lower glucose concentrations in 12 and as reduced insulin administration (with similar glucose concentrations) in 3. Owing to heterogeneity, meta-analysis was possible only for the outcome of hypoglycaemia. This revealed an incidence of 7.4% in those receiving incretin-based therapies and 6.8% in comparator groups (*P* = 0.94).

**Conclusions:**

In small, single-centre studies, incretin-based therapies lowered blood glucose and reduced insulin administration without increasing the incidence of hypoglycaemia.

**Trial registration:**

PROSPERO, CRD42017071926.

**Electronic supplementary material:**

The online version of this article (10.1186/s13054-018-2197-4) contains supplementary material, which is available to authorized users.

## Background

Hyperglycaemia occurs frequently in the peri-operative period and during critical illness, even in patients without a history of diabetes mellitus [1–3]. Usual management of hyperglycaemia in these settings primarily involves intravenous infusions of insulin, with the dose titrated according to intermittent measurement of blood glucose [4]. This strategy is somewhat complicated and labour–intensive, and it increases the risk of hypoglycaemia and glycaemic variability, which are both associated with adverse outcome [3, 5–10].

The incretin effect is the physiological phenomenon observed following the ingestion of glucose, which results in endogenous insulin secretion almost two-fold greater than after a comparable intravenous glucose load [11]. This process is attributed to the enterohormones glucagon-like peptide 1 (GLP-1) and glucose-dependent insulinotropic peptide (GIP) that have insulinotropic and glucagonostatic properties [12]. The insulinotropic response is glucose-dependent, meaning that even when GLP-1 and GIP are administered in pharmacological doses, there is negligible risk of hypoglycaemia [12].

GLP-1 and GIP are rapidly metabolised by the enzyme dipeptidyl peptidase IV (DPP-IV) [12]. Accordingly, incretin-based therapies necessitate a continuous infusion of either exogenous GLP-1 or GIP, administration of a DPP-IV-resistant receptor agonist (GLP-1 receptor agonists, first-in-class drug exenatide), or a DPP-IV antagonist that increases endogenous GLP-1 and GIP concentrations (first-in-class drug sitagliptin) [12]. All currently available and applicable drugs are named in Additional file 1.

GLP-1 receptor agonists and DPP-IV inhibitors are now established therapies for the management of patients with type 2 diabetes mellitus (T2DM) [13]. The efficacy and safety profiles of incretin-based therapies have fostered enthusiasm for use of these agents as adjuncts or alternatives to insulin for glycaemic control in the operating room and intensive care unit (ICU). The purpose of this systematic review was to evaluate the safety and efficacy of incretin therapies for glucose control in the operating room and ICU.

## Methods

This systematic review was prospectively registered in the PROSPERO database (PROSPERO identifier CRD42017071926) and conducted according to the Preferred Reporting Items for Systematic Reviews and Meta-Analyses (PRISMA) guidelines [14].

### Eligibility criteria

Studies eligible for inclusion were prospective randomised controlled trials using an incretin-based therapy in the operating room and/or the ICU. Studies published in any language and without publication date restriction were considered. Paediatric, animal and observational studies were excluded.

### Search strategy

We performed an unrestricted electronic database search of the MEDLINE, Cochrane Library and Embase databases from their inception to 13 February 2018. Our search included terms to specify the intervention (incretin therapy), setting (peri-operative and ICU care) and study type (prospective randomised controlled trials). Searches included synonyms and combinations of the following terms: ‘operating room’, ‘OR’, ‘peri-operative period’, ‘ICU’, ‘critical care’, ‘incretin therapy’, ‘GLP-1’, ‘GIP’ and ‘DPP-IV inhibitor’, as well as generic names of the currently marketed forms of these medications. Our complete search terms and methodology are available as additional material (*see* Additional file 1) and accessible via PROSPERO. Reference lists of retrieved papers were also reviewed for potentially eligible studies not captured in the primary search. We defined no specific comparator for any intervention.

### Study selection

After deletion of duplicate studies, two investigators (AHH, MPP) screened all titles and abstracts using Rayyan [15]. Relevant studies were then evaluated in full text for eligibility, with any conflicts resolved by a third investigator (JH). The authors of conference abstracts and published protocols without subsequent full texts were contacted to request the data and/or manuscript.

### Risk-of-bias assessment

Two authors independently assessed the quality of the research methodology of all randomised controlled trials using the Cochrane Collaboration’s Risk of Bias Tool [16].

### Data extraction

We extracted data including study characteristics (author, publication year, country, design, funding source and sample size), setting (operating room, ICU, post-cardiac surgery), patient characteristics (demographics) and intervention and comparator parameters (incretin therapy, route, dose and duration, as well as additional treatments). We did not predefine primary outcomes in this scoping exploratory systematic review; all reported outcomes were recorded and summarised if reported across multiple studies. Owing to the expected heterogeneity of interventions, comparators, settings and outcomes, we did not plan a meta-analysis of outcomes. Owing to the frequency with which hypoglycaemia was reported across studies, we decided to retrospectively perform a meta-analysis of this outcome. This was not feasible for all other outcomes.

### Statistical analysis

For data extraction and meta-analysis, we used Review Manager version 5.3 (The Cochrane Collaboration, The Nordic Cochrane Centre, Copenhagen, Denmark). We used a random effects model because of expected clinical heterogeneity between trials. Results of the meta-analysis were expressed as Mantel-Haenszel odds ratios with 95% CIs because of the dichotomous outcome. As markers for inter-trial heterogeneity, we used τ^2^, χ^2^ and *I*^2^ statistics.

## Results

**Fig. 1:**
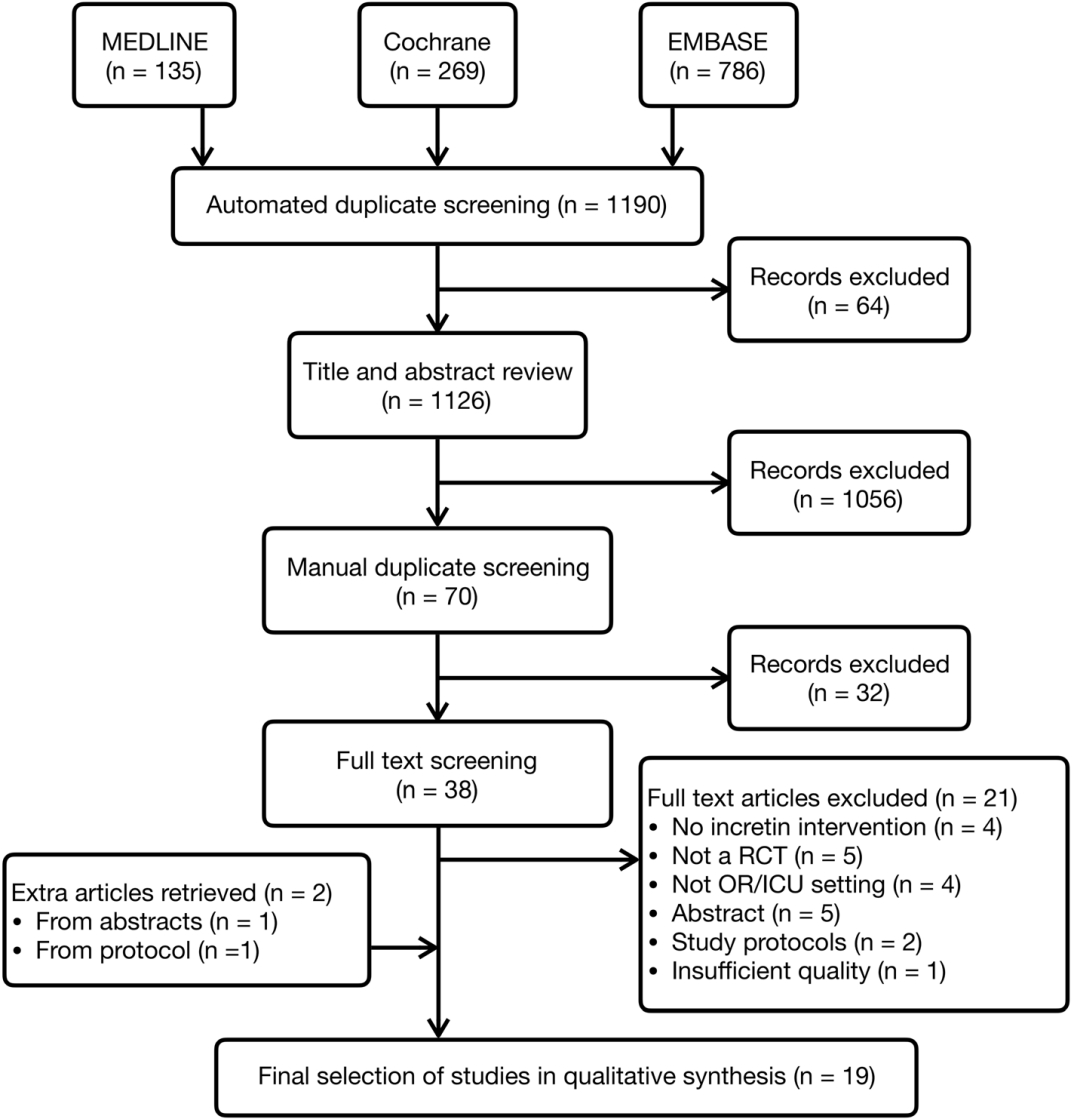
Preferred Reporting Items for Systematic Reviews and Meta-Analyses (PRISMA) flow diagram. *ICU* Intensive care unit, *OR* Operating room, *RCT* Randomised controlled trial

Our search yielded 1126 citations, and after elimination of duplicates, abstracts and full texts, 19 studies were included in this systematic review (Fig. 1).

### Study characteristics

Characteristics of the included studies are summarised in Table 1, including setting of care, duration, type and dose of intervention, and reported outcomes [17–34]. In total, 1410 patients participated in these studies, of whom 988 were known to have T2DM. All studies recruited patients in a single centre. Comparator groups included placebo or combinations of intravenous or subcutaneous insulin.

**Table 1:** Study characteristics

Author, year	Participants, setting, *n*	DM, *n* (%)	Intervention duration	Intervention, dose, *n*	Comparator, *n*	Standard glycaemic therapy	Outcome parameters
Besch, 2017 [19]	CABG, OR + ICU *n* = 104	22 (21%)	48 h	Exenatide IV 25 ng/min *n* = 53	Standard glycaemic therapy *n* = 51	Continuous insulin IV + bolus regimen	Glycaemia *Insulin administration, complications, LoS*
Brackbill, 2012 [20]	CABG, ward, *n* = 62	62 (100%)	4 d	Sitagliptin PO 100 mg q.d. *n* = 30	Placebo *n* = 32	Basal bolus insulin SC regimen	Glycaemia *LoS*
Deane, 2009 [21]	Mechanically ventilated, ICU *n* = 7	0 (0%)	240 min	GLP-1 IV 1.2 pmol/kg/min *n* = 7	Placebo *n* = 7	None	Glycaemia *Insulinaemia, Glucagon, GLP-1*
Deane, 2010 [22]	Mechanically ventilated, ICU *n* = 25	0 (0%)	360 min	GLP-1 IV 1.2 pmol/kg/min *n* = 25	Placebo *n* = 25	None	Glycaemia *Gastric emptying, glucose absorption, Insulinaemia, Glucagon*
Deane, 2011 [23]	Mechanically ventilated, ICU *n* = 11	11 (100%)	240 min	GLP-1 IV 1.2 pmol/kg/min *n* = 11	Placebo *n* = 11	None	Glycaemia *Insulinaemia, C-peptide, glucagon, FFA*
Galiatsatos, 2014 [24]	Surgical/burn, ICU *n* = 18	9 (50%)	72 h	GLP-1 IV 1.5 pmol/kg/min *n* = 9	Saline *n* = 9	Intensive insulin treatment protocol	Glycaemia *Insulin administration, glucagon, C-peptide, CV medication*
Garg, 2017 [35]	In hospital, ward (74% surgical) *n* = 66	66 (100%)	5 d	Saxagliptin PO 5 mg q.d. *n* = 33	Basal bolus insulin SC regimen *n* = 33	Corrective insulin bolus regimen	Glycaemia *Insulin administration, Treatment failure, LoS*
Holmberg, 2014 [25]	CABG, OR *n* = 62	12 (19%)	390 min	Exenatide IV 43 ng/min *n* = 21	RIPC *n* = 20 / Placebo *n* = 21	Unknown	Cardiac enzymes *Complications, LoS*
Kar, 2015 [26]	Mechanically ventilated, ICU *n* = 20	0 (0%)	300 min	GIP IV 4 pmol/kg/min *n* = 20	Placebo *n* = 20	None	Glycaemia *Gastric emptying, glucose absorption, insulinaemia*
Kohl, 2014 [27]	CABG, OR *n* = 77	11 (14%)	72 h	GLP-1 IV 1.5 pmol/kg/min *n* = 37	Placebo *n* = 40	Continuous insulin IV + bolus regimen	Glycaemia *Insulinaemia, glucagon, GLP-1, cortisol, FFA.*
Lee, 2013 [28]	Mechanically ventilated, ICU *n* = 20	0 (0%)	300 min	GIP IV 4 pmol/kg/min *n* = 20	Standard glycaemic therapy *n* = 20	GLP-1 IV 1.2 pmol/kg/min (300 min)	Glycaemia *Insulinaemia, glucagon, GLP-1, GIP,*
Lipš, 2017 [17]	CABG, OR *n* = 38	26 (68%)	72 h	Exenatide IV 20 ng/min *n* = 19	Placebo *n* = 19	Intensive insulin treatment protocol	Glycaemia *Echocardiography, CV medications, complications*
Meier, 2004 [29]	Major surgery, ward *n* = 8	100 (100%)	8 h	GLP-1 IV 1.2 pmol/kg/min *n* = 8	Placebo *n* = 8	None	Glycaemia *Insulinaemia, C-peptide, glucagon, GLP-1*
Miller, 2017 [30]	Mechanically ventilated, ICU *n* = 12	0 (0%)	270 min	GLP-1 IV 1.2 pmol/kg/min *n* = 12	Placebo *n* = 12	None	Glycaemia *Glucose absorption*
Müssig, 2008 [31]	CABG, ICU *n* = 20	100 (100%)	12 h	GLP-1 IV 3.6 pmol/kg/min *n* = 10	Continuous insulin IV *n* = 10	Corrective insulin bolus regimen	Glycaemia *Insulin administration, haemodynamics*
Pasquel, 2017 [32]	In hospital, ward (16% surgical) *n* = 277	100 (100%)	10 d	Sitagliptin PO 100 mg q.d. *n* = 138	Bolus insulin regimen *n* = 139	Basal (glargine) insulin regimen	Glycaemia *Insulin administration, complications, treatment failure*
Polderman, 2018 [18]	Surgical, OR *n* = 150	100 (100%)	2 d	Liraglutide SC 0.6 mg + 1.2 mg *n* = 44	GIK infusion *n* = 53/Bolus insulin algorithm *n* = 53	Bolus insulin treatment algorithm	Glycaemia *Insulin administration,* *Potassium, nausea, complications*
Sokos, 2007 [34]	CABG, OR *n* = 20	5 (25%)	60 h	GLP-1 IV 1.5 pmol/kg/min *n* = 10	Standard insulin therapy *n* = 10	Standard insulin therapy	Glycaemia *LVEF, haemodynamics*
Umpierrez, 2014 [33]	In hospital, ward (45% surgical) *n* = 90	100 (100%)	10 d	Sitagliptin PO 100 mg q.d. *n* = 27 / Sitagliptin + basal insulin *n* = 29	Basal bolus insulin regimen *n* = 26	Correction bolus insulin regimen	Glycaemia *Insulin administration, complications, treatment failure*

*Abbreviations: b.i.d.* Twice per day, *CABG* Coronary artery bypass grafting, *CV* Cardiovascular, *d* Days, *DM* Diabetes mellitus, *FFA* Free fatty acids, *GIK* Glucose-insulin-potassium infusion, *GIP* Gastric inhibitory polypeptide, *GLP-1* Glucagon-like peptide-1, *h* Hours, *ICU* Intensive care unit, *IV* Intravenously, *LoS* Length of stay, *min* Minutes, *LVEF* Left ventricular ejection fraction, *OR* Operating room, *PO* By mouth, *q.d* Once per day, *RIPC* Remote ischaemic preconditioning, *SC* Subcutaneous

All secondary outcomes are in italics

### Risk of bias

**Fig. 2:**
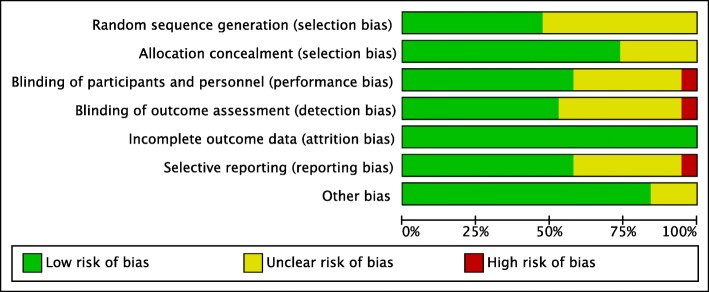
Review authors’ judgements about each risk-of-bias item presented as percentages across all included studies. *Green* = low risk of bias; *yellow* = unclear risk of bias; *red* = high risk of bias

**Fig. 3:**
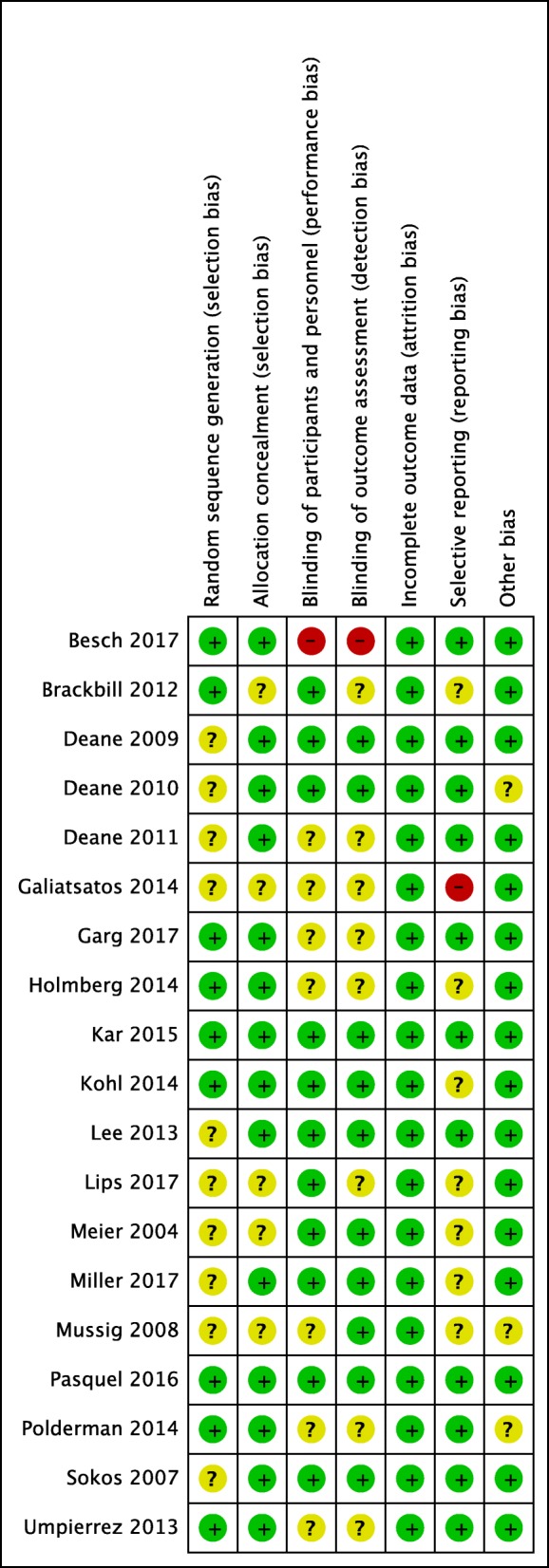
Review authors’ judgements about each risk-of-bias item for each included study. *Green* = low risk of bias; *yellow* = unclear risk of bias; *red* = high risk of bias

A summary of the risk of bias in the included studies is presented in Figs. 2 and 3. Randomisation sequence generation was often briefly described and therefore assessed as unclear. Allocation concealment carried a low risk of bias in most studies and was scored as unclear only if it remained unmentioned in the manuscript. Most trials were blinded and adequately described as such. In some trials the intervention was not blinded; however, if the primary outcome was a measurable physiological variable (e.g., glucose), a low risk of bias was ascribed. Only one trial was deemed to have a high risk of bias owing to both open-label administration of study drug and an outcome measure (insulin administration) that has the capacity to be influenced by the knowledge of treatment allocation [19]. With limited numbers of patients per study and short follow-up periods for the main outcome parameters, attrition bias was deemed low in all studies. Because most studies reported similar outcomes (Table 1), the risk of selective reporting between studies was considered low. The majority of studies had registered protocols demonstrating consistent reporting of outcomes, and in only one case was there a discrepancy between reported and registered outcomes [24]. Other potential sources of bias identified were an early termination due to slow enrolment [18], deviation from baseline reporting for some outcomes [22] and one study published as a letter to the editor with consequent brief reporting and unclear identification of sources of bias [31].

### Efficacy of intervention

A measurement of glycaemic control was reported as the primary outcome in 17 of 19 included studies. We summarise all primary outcomes in Table 2.

**Table 2:** Summary of main outcomes of all included studies

Author, year	Main outcome	Result
Meier, 2004 [29]	GLP-1 IV lowered mean glucose levels	*+*
Sokos, 2007 [34]	GLP-1 IV reduced peri-operative glucose levels	*+*
Müssig, 2008 [31]	GLP-1 IV reduced insulin administration with comparable glycaemic control	+
Deane, 2009 [21]	GLP-1 IV lowered mean post-prandial glucose levels	+
Deane, 2010 [22]	GLP-1 IV lowered mean post-prandial glucose levels	+
Deane, 2011 [23]	GLP-1 IV lowered mean post-prandial glucose levels	+
Galiatsatos, 2014 [24]	GLP-1 IV did not lower mean glucose levels	–
Kohl, 2014 [27]	GLP-1 IV lowered mean glucose levels	*+*
Miller, 2017 [30]	GLP-1 IV reduced intestinal glucose absorption	*+*
Kar, 2015 [26]	GIP IV did not lower mean glucose levels	*–*
Lee, 2013 [28]	GIP IV did not lower mean glucose levels	*–*
Polderman, 2018 [18]	Liraglutide SC reduced post-operative glucose levels	*+*
Holmberg, 2014 [25]	Exenatide IV did not lower post-operative cardiac enzymes	–
Besch, 2017 [19]	Exenatide IV did not increase number of patient that spend > 50% in target range	*–*
Lipš, 2017 [17]	Exenatide IV did not improve left ventricular ejection fraction	*–*
Garg, 2017 [35]	Saxagliptin PO resulted in similar glucose levels compared with basal bolus insulin	*+*
Pasquel, 2017 [32]	Sitagliptin PO as adjunct to basal insulin resulted in similar glucose levels compared with bolus insulin	*+*
Umpierrez, 2014 [33]	Sitagliptin PO resulted in similar glucose levels compared with basal bolus insulin	–
Brackbill, 2012 [20]	Sitagliptin PO did not lower the mean postoperative glucose levels	*–*

*Abbreviations: GIP* Gastric inhibitory polypeptide, *GLP-1* Glucagon-like peptide-1, *IV* Intravenously, *PO* By mouth, *SC* Subcutaneous

+ = study positive for primary outcome, − = study negative for primary outcome

#### Intra-operative glucose lowering

A number of studies assessed the effect of GLP-1 receptor stimulation as an adjunct to standard insulin therapy during cardiac surgery. The first of these randomised 20 patients to a continuous intravenous infusion of GLP-1 (1.5 pmol kg^− 1^ min^− 1^) or placebo, commencing 12 h pre-operatively and continuing for 48 h post-operatively. GLP-1 resulted in lower mean glucose in the pre- and peri-operative periods, with nearly half the insulin administered to achieve comparable glycaemic control in the post-operative periods [34]. In 77 patients undergoing elective cardiac surgery, using the same dose of intravenous GLP-1 infused intra-operatively, Kohl and colleagues reported that mean blood glucose values were 0.68 mmol L^− 1^ lower for subjects receiving GLP-1 compared with those receiving placebo (95% CI, 0.13–1.22 mmol L^− 1^; *P* = 0.015) [27]. Lipš and colleagues randomised 38 patients with decreased left ventricular function undergoing coronary artery bypass grafting (CABG) to a 72-h infusion of intravenous exenatide (20 ng min^− 1^) or placebo as an adjuvant to standard insulin therapy [17]. Patients receiving exenatide demonstrated lower peri-operative mean blood glucose (6.4 ± 0.5 vs. 7.3 ± 0.8 mmol/L; *P* < 0.001) and a greater percentage of time in the target range of 4.5–6.5 mmol/L (54.8% ± 14.5% vs. 38.6% ± 14.4%; *P* = 0.001). In a similar study of 104 patients undergoing elective CABG, Besch and colleagues did not observe a statistical difference in the glycaemic outcome of interest (time in target range) between intravenous exenatide (25 ng min^− 1^) and placebo; however, exenatide was insulin-sparing with a longer time to commencement of insulin and significantly less insulin administered [19]. Polderman and colleagues compared pre- and intra-operative subcutaneous liraglutide (0.6 mg + 1.2 mg) (a GLP-1 receptor agonist) with an intravenous glucose-insulin-potassium infusion and an insulin bolus regimen [18]. Median plasma glucose 1 h post-operatively was lower in the liraglutide group (6.6 mmol L^− 1^) than in both the continuous insulin infusion (7.5 mmol L^− 1^) and insulin bolus (7.6 mmol L^− 1^) groups (*P* = 0.015). In this study, liraglutide showed an insulin-sparing effect, with fewer episodes of insulin administration and reduced total insulin administration.

#### Post-operative glucose lowering

In their vanguard study, Meier and colleagues randomised eight patients with T2DM who had undergone major surgery within the preceding week to 8-h infusions of intravenous GLP-1 (1.2 pmol kg^− 1^ min^− 1^) and placebo in a cross-over fashion [29]. GLP-1 ‘normalised’ blood glucose (fasting < 7 mmol/L) in the cohort within 150 min, whereas patients remained hyperglycaemic (> 8 mmol/L) in the control arm [29]. In a further study of post-operative glycaemic control in T2DM, Müssig and colleagues randomised patients to GLP-1 (3.6 pmol kg^− 1^ min^− 1^) or standard intravenous insulin in the 12 h following CABG [31]. Glycaemic control was comparable between groups; however, the GLP-1 cohort had significantly less insulin administered during the first 6 h following surgery [31].

Studies assessing the efficacy of the oral DPP-IV inhibitor sitagliptin for post-operative glycaemic control in patients with T2DM have reported varied results. In the study by Brackbill and colleagues the post-CABG addition of sitagliptin (100 mg once daily) to standard subcutaneous basal insulin and regular oral hypoglycaemic agents did not result in any difference in glycaemia or insulin administration [20]. Two related studies on the ward, one [33] a pilot preceding a larger trial [32], which included both medical and surgical patients (Table 1), assessed sitagliptin (100 mg once daily) as an adjunct to a basal insulin when compared with a standard basal bolus insulin regimen. The primary outcome of the larger trial was non-inferiority of mean blood glucose. Sitagliptin group was non-inferior to standard care and was associated with less total daily insulin requirement (24 ± 16 U/d vs. 34 ± 20 U/d; *P* < 0.001) [32]. Garg and colleagues compared the oral DPP-IV inhibitor saxagliptin (5 mg once daily) with basal bolus insulin in a non-critically ill population of hospitalised patients with T2DM, predominantly in the post-operative period [35]. Saxagliptin was non-inferior to basal bolus insulin for glycaemic control as determined by the daily mean blood glucose (primary outcome), with saxagliptin treatment causing less glycaemic variability [35].

#### Intensive care unit

Deane and colleagues have assessed continuous intravenous infusions of GLP-1 in a series of cross-over trials in heterogeneous cohorts of mechanically ventilated patients [21–23, 30]. At a dose of 1.2 pmol kg^− 1^ min^− 1^ infused over 270 to 330 min, GLP-1 reduced the glycaemic response to small intestinal nutrient delivery in patients with T2DM [23] and to intra-gastric and small intestinal nutrient delivery in patients not known to have T2DM [21, 22, 30]. Enteral nutrient-stimulated hyperglycaemia was attenuated but not suppressed completely at this dose, with the glucose-lowering effect more prominent in those patients without a history of diabetes. This group also evaluated the glycaemic effect of intravenous infusions of GIP during intra-gastric and small intestinal nutrient administration in mechanically ventilated patients, and, in contrast to the profound glucose-lowering effect of GIP in health, they reported no glucose-lowering effect when GIP was given as stand-alone therapy or added to GLP-1 [26, 28]. Galiatsatos and colleagues compared an extended intravenous GLP-1 infusion (1.5 pmol kg^− 1^ min^− 1^ for 72 h) with placebo as an adjunct to intensive insulin therapy in critically ill surgical patients. They reported no difference in mean blood glucose or insulin use between groups, but substantially less glycaemic variability (given by the co-efficient of variation of mean glucose) was observed in the GLP-1 cohort [24].

### Hypoglycaemia

Data regarding hypoglycaemia are summarised in Table 3. The threshold to diagnose moderate hypoglycaemia ranged from < 2.8 to < 4.0 mmol/L. The incidence of moderate hypoglycaemia in the incretin arm varied from zero to 17%, except for one outlier with a reported incidence of 36% (8 of 23 patients) [25]. In the latter trial intravenous exenatide was infused at double the dose of subsequent trials, and it is unclear whether insulin was concurrently administered [25]. Meta-analysis revealed no difference in incidence of hypoglycaemia (incretin-based therapy 36 of 484 [7.4%] vs. comparator 36 of 540 [6.7%], *P* = 0.96). Of note, incretin-based therapies were administered with insulin in 10 of the 14 studies reporting hypoglycaemia (Table 1).

**Table 3:** Analysis of hypoglycaemia in reported studies

Author, year	Threshold to define hypoglycaemia	Incretin		Comparator		Weight	Odds ratio M-H, random, 95% CI	*P* value	Odds ratio M-H, random, 95% CI
		*n*	group	*n*	group				
Besch, 2017 [19]	3.3 mmol L^− 1^	2	53	1	51	8.1%	1.96 [0.17, 22.32]	0.58	
Brackbill, 2012 [20]	3.3 mmol L^− 1^	5	30	2	32	12.9%	3.00 [0.54, 16.81]	0.06	
Deane, 2010 [22]	3.0 mmol L^− 1^	0	25	0	25		Not estimable	1	
Galiatsatos, 2014 [24]	2.8 mmol L^− 1^	1	9	3	9	7.8%	0.25 [0.02, 3.04]	0.58	
Garg, 2017 [35]	3.9 mmol L^− 1^	1	33	1	33	5.7%	1.00 [0.06, 16.69]	1	
Holmberg, 2014 [25]	4.0 mmol L^− 1^	8	21	0	41	6.1%	52.26 [2.83, 966.6]	0.003	
Kar, 2015 [26]	Not stated	0	24	0	24		Not estimable	1	
Kohl, 2014 [27]	3.8 mmol L^− 1^	0	37	0	40		Not estimable	1	
Lipš, 2017 [17]	3.3 mmol L^− 1^	2	19	4	19	11.9%	0.44 [0.07, 2.76]	0.12	
Meier, 2004 [29]	4.0 mmol L^−1^	0	8	0	8		Not estimable	1	
Müssig, 2008 [31]	Not stated	0	10	0	10		Not estimable	1	
Pasquel, 2017 [33]	3.9 mmol L^−1^	13	138	17	139	24.7%	0.75 [0.35, 1.60]	0.45	
Polderman, 2018 [18]	4.0 mmol L^−1^	1	44	5	106	9.5%	0.47 [0.05, 4.14]	0.26	
Sokos, 2007 [34]	3.3 mmol L^−1^	1	10	2	10	7.4%	0.44 [0.03, 5.88]	0.39	
Umpierrez, 2014 [33]	3.9 mmol L^−1^	3	56	2	26	11.8%	0.68 [0.11, 4.33]	0.86	
Total (95% CI)			484		540	100%	0.97 [0.47, 2.02]	0.94	
Total events		37		37					

*M-H* Mantel-Haenszel

Heterogeneity: Tau^2^ = 0.39, Chi^2^ = 12.94, *df* = 9 (*P* = 0.17); *I*^2^ = 30%

Test for overall effect: *Z* = 0.08 (*P* = 0.94)

### Non-glycaemic effects

Owing to the heterogeneity of definitions and infrequency of reporting of non-glycaemic end-points, quantitative analysis of these data was not possible. Plasma insulin and glucagon concentrations were reported in eight studies [21–24, 26–28]. GLP-1 was reported to increase plasma insulin levels [23, 29] or insulin/glucose ratios [21, 22] in enterally fed critically ill and post-operative patients. However, this insulinotropic effect was not observed in studies that sampled blood intra-operatively in fasted patients [27, 34]. The effect of GLP-1 on glucagon concentration was similarly heterogeneous, with several studies reporting a glucagonostatic effect [24, 29, 34] and others reporting no difference [21, 22, 27]. The addition of GIP to a GLP-1 regimen in critically ill patients did not have an additional insulinotropic effect [28], and GIP as a sole agent was not shown to have an effect on plasma insulin or glucagon concentrations in critically ill patients [26].

In the critically ill, GLP-1 slows gastric emptying when emptying is relatively normal, but it appears to have minimal effect when emptying is already delayed [22], whereas GIP appears to have no effect on gastric motility [26]. Similarly, GLP-1 delayed enteral glucose absorption, even when nutrient was delivered directly into the small intestine [23, 30], whereas GIP had no effect [26].

Five studies compared the cardiovascular effects of GLP-1 or a GLP-1 receptor agonist with placebo [17, 24, 25, 31, 34]. In these studies there were no differences in cardiac enzymes [17, 25], echocardiographic measurements of left ventricular function [17, 34], haemodynamic parameters (heart rate, mean arterial pressure, pulmonary artery diastolic pressure) [31, 34] or vasoactive medication requirement [17, 24, 25, 31].

There was no difference in the incidence of post-operative nausea and vomiting in studies comparing placebo with intravenous exenatide [19], oral sitagliptin [32] and subcutaneous liraglutide [18]. However, pre-operative nausea was more common when subcutaneous liraglutide was administered the night before surgery (13% vs. 0%, *P* = 0.007, *n* = 150) [18]. Incretin-based therapies have not been reported to increase post-operative complications or serious adverse events [17–19, 25, 32].

### Diabetes mellitus

Eight studies were performed exclusively in patients with T2DM [18, 20, 23, 29, 31–33, 35], five studies in patients without T2DM [21, 22, 26, 28, 30] and a further six studies in mixed cohorts of patients with and without T2DM (Table 1) [17, 19, 24, 25, 27, 34]. None of the studies recruiting mixed populations reported subgroup analyses according to diabetic status. Owing to the heterogeneity of interventions and outcomes, it was not possible to draw meaningful conclusions on the effects of incretins in patients with T2DM compared with those without.

## Discussion

We systematically reviewed all randomised controlled trials of incretin-based interventions performed in the operating room and/or ICU setting and identified 19 studies which included 1410 patients in aggregate. Most studies reported a reduction in blood glucose or glycaemic variability when incretin-based therapies were used as a sole agent and/or a decrease in insulin administration when used as adjuvant therapy. Incretin-based therapies did not significantly reduce the incidence of hypoglycaemia. Incretin-based therapies did appear to attenuate glycaemic variability, although the latter was infrequently reported.

A number of studies attempted to delineate mechanisms underlying glucose-lowering in this cohort. The recognised insulinotropic effect of GLP-1 was consistently demonstrated in enterally fed patients, whereas glucagonostasis was less reliably reported. In small, single-centre studies, exogenous GLP-1 slowed gastric emptying in the setting of normal gastric motility and delayed intestinal glucose absorption, both of which likely contribute to attenuating nutrient stimulated hyperglycaemia [22, 30].

Although compliance with GLP-1 receptor agonists is relatively good in ambulant patients with T2DM, the primary reason for discontinuation of therapy is gastrointestinal discomfort, particularly nausea and vomiting [36, 37]. Critically ill and post-operative patients are at increased risk of nausea and vomiting, and it is therefore somewhat surprising that only three of the studies reported this side effect. Notwithstanding the relatively small number of patients studied, it is reassuring that incretin therapy did not appear to further increase the risk of post-operative nausea and vomiting.

Large trials in ambulant patients with T2DM have reported beneficial cardiovascular effects with GLP-1 receptor agonists [38–40]. This signal is supported by preliminary animal and observational human data identifying potential cardioprotective properties of incretin-based therapies [41, 42]. This provides a persuasive rationale for the use of GLP-1 in the setting of cardiac surgery. In murine models, GLP-1 decreases ischaemia-induced myocardial damage [41], and in patients with heart failure, exogenous GLP-1 has been associated with improvements in left ventricular ejection fraction, myocardial oxygen uptake and 6-min walk distance [42]. However, the most recent trial in patients with diabetes and heart failure observed no difference in time to death or rehospitalisation for heart failure [43]. None of the studies included in this review reported any differences in acute indices of cardiac performance between incretin-based therapies and control.

### Strengths and limitations

Strengths of this systematic review include the structured search, complete retrieval of the identified research and validated methods in accordance with the PRISMA statement. However, there are some limitations. We found marked clinical heterogeneity between the studies, including the dose and type of incretin therapy and duration of intervention, ranging from 4 h to 10 days. In addition, there were substantial differences in the glycaemic control strategies of the control arms, ranging from blinded placebo to open-label intravenous insulin. The broad scope of this review revealed a marked heterogeneity in the populations studied, which included patients undergoing elective cardiac surgery, ward surgical patients and mechanically ventilated critically ill patients. Furthermore, there were trials performed exclusively in patients with pre-existing diabetes, whereas in other trials patients with pre-existing diabetes were excluded, and still others included both groups of patients. Inferences should therefore be circumspect because it is increasingly recognised that hyperglycaemia does not represent the same insult to all patients and may be modified by patients’ pre-morbid glycaemic control [44]. It should be noted, however, that the majority of included patients were diagnosed with DM. Although all of the studies assessed ‘glycaemic control’, there was substantial variation in the outcomes reported, such that meta-analysis was possible only on the variable of hypoglycaemia. Finally, most studies were small, single-centre trials and thus underpowered to detect differences in clinical and patient-centred outcomes and safety end-points.

### Future directions

Taken together, these data signal the potential for incretin-based therapies, particularly GLP-1-based regimens, as effective glucose-lowering agents with a relatively low incidence of hypoglycaemia. However, owing to the limitations of the original studies, it is not possible to draw definitive conclusions regarding the role of incretin therapies in the operating room and ICU. Future studies are required to determine (1) the population most likely to benefit; (2) optimal dosing regimens, including the role for combination therapy with insulin; and (3) clinical efficacy and safety outcomes.

## Conclusions

Incretin-based therapies represent a promising, novel approach to glucose control in the peri-operative period and during critical illness, with a low risk of hypoglycaemia. Further studies with larger sample sizes [45] are required to determine the optimal agent and dosing regimen and effects on patient-centred outcomes.

## Additional file

Additional file 1: Methodology of systematic review on incretins in peri-operative and intensive care (PDF 88 kb)

## Abbreviations

CABG: Coronary artery bypass grafting
CV: Cardiovascular
DM: Diabetes mellitus
DPP-IV: Dipeptidyl peptidase IV
FFA: Free fatty acids
GIK: Glucose-insulin-potassium infusion
GIP: Glucose-dependent insulinotropic peptide
GLP-1: Glucagon-like peptide 1
ICU: Intensive care unit
IV: Intravenously
LoS: Length of stay
LVEF: Left ventricular ejection fraction
PO: By mouth
RIPC: Remote ischaemic preconditioning
SC: Subcutaneous
T2DM: Type 2 diabetes mellitus
