# Supplementary material for: Systematic review of incretin therapy during peri-operative and intensive care
Source: Crit Care. 2018 Nov 14;22:299. doi: 10.1186/s13054-018-2197-4 (PMC6236901; doi:10.1186/s13054-018-2197-4)

Methodology of systematic review on incretins in perioperative and intensive care

1. Pubmed Search (pubmed.org)

1.1. Search terms:

((((("sitagliptin"[tiab] OR "vildagliptin"[tiab] OR "saxagliptin"[tiab] OR "linagliptin"[tiab] OR "gemigliptin"[tiab] OR "anagliptin"[tiab] OR "teneligliptin"[tiab] OR "alogliptin"[tiab] OR "dutogliptin"[tiab] OR "trelagliptin"[tiab] OR "omarigliptin"[tiab] OR "dulaglutide"[tiab] OR "exenatide"[tiab] OR "liraglutide"[tiab] OR "GLP-1"[tiab] OR "glucagon-like peptide"[tiab] OR "incretin"[tiab]) OR ("Glucagon-Like Peptides"[Mesh] OR "Liraglutide"[Mesh] OR "Incretins" [Pharmacological Action] OR "Dipeptidyl-Peptidase IV Inhibitors"[Mesh]))*

AND

((preoperat*[tiab] OR perioperat*[tiab] OR pre-operat*[tiab] OR peri-operat*[tiab] OR surgery*[tiab] OR "intensive care"[tiab] OR "critical care"[tiab] OR "critical illness"[tiab] OR ("critical"[tiab] AND "illness"[tiab]) OR "critical illness"[tiab] OR ("critically"[tiab] AND "ill"[tiab]) OR "critically ill"[tiab]) OR ("Perioperative Care"[Mesh] OR "Critical Care"[Mesh] OR "Intensive Care Units"[Mesh])))))

AND

(("Clinical Trial" [PT:NoExp] OR "clinical trial, phase i"[pt] OR "clinical trial, phase ii"[pt] OR "clinical trial, phase iii"[pt] OR "clinical trial, phase iv"[pt] OR "controlled clinical trial"[pt] OR "multicenter study"[pt] OR "randomized controlled trial"[pt] OR "Clinical Trials as Topic"[mesh:noexp] OR "clinical trials, phase i as topic"[MeSH Terms:noexp] OR "clinical trials, phase ii as topic"[MeSH Terms:noexp] OR "clinical trials, phase iii as topic"[MeSH Terms:noexp] OR "clinical trials, phase iv as topic"[MeSH Terms:noexp] OR "controlled clinical trials as topic"[MeSH Terms:noexp] OR "randomized controlled trials as topic"[MeSH Terms:noexp] OR "early termination of clinical trials"[MeSH Terms:noexp] OR "multicenter studies as topic"[MeSH Terms:noexp] OR “Double-Blind Method”[Mesh] OR ((randomised[TIAB] OR randomized[TIAB]) AND (trial[TIAB] OR trials[tiab])) OR ((single[TIAB] OR double[TIAB] OR doubled[TIAB] OR triple[TIAB] OR tripled[TIAB] OR treble[TIAB] OR treble[TIAB]) AND (blind*[TIAB] OR mask*[TIAB])) OR ("4 arm"[tiab] OR "four arm"[tiab])))

1.2. Filters:

None

1.3. Date Run:

13-2-2018

1.4. Results:

135

1.5. Actions:

Exported as FILE, Format: Medline from Pubmed

Saved: Search Pubmed SR GLP1 OK IC 13-2-2018.txt

Added to Endnote: Import file, select file, option: Pubmed (NLM)

2. Cochrane Search (onlinelibrary.wiley.com)

2.1. Search terms:

glp or glp-1 or glucagon-like or liraglutide or incretin* or dulaglutide or exenatide or sitagliptin or vildagliptin or saxagliptin or linagliptin or gemigliptin or anagliptin or teneligliptin or alogliptin or dutogliptin or trelagliptin or omarigliptin:ti,ab,kw (Word variations have been searched)

and

operation or surgery or preoperat or perioperat* or pre-operat* or peri-operat* or anesth* or anaesth* or "intensive care" or "critical care":ti,ab,kw (Word variations have been searched)*

2.2. Filters:

None

2.3. Date Run:

13-2-2018

2.4. Results:

269

2.5. Actions:

Exported as File, Format: PC

Saved: Search Cochrane SR GLP1 OK IC 13-2-2018 file 1.txt + Cochrane SR GLP1 OK IC 13-2-2018 file 2

Added to Endnote: Import file, select file, option: Cochrane (Wiley)

3. Embase Search (Ovidsp.tx.ovid.com)

3.1. Search terms:

1 ("clinical trial" or "clinical trial, phase i" or "clinical trial, phase ii" or clinical trial, phase iii or clinical trial, phase iv or controlled clinical trial or "multicenter study" or "randomized controlled trial").pt. or double-blind method/ or clinical trials as topic/ or clinical trials, phase i as topic/ or clinical trials, phase ii as topic/ or clinical trials, phase iii as

topic/ or clinical trials, phase iv as topic/ or controlled clinical trials as topic/ or randomized controlled trials as topic/ or early termination of clinical trials as topic/ or multicenter studies as topic/ or ((randomi?ed adj7 trial) or (controlled adj3 trial*) or (clinical adj2 trial*) or ((single or doubl* or tripl* or treb*) and (blind* or mask*))).ti,ab,kw. or ("4 arm" or "four arm").ti,ab,kw.*

2 exp glucagon like peptide 1 derivative/ or exp glucagon like peptide 1/ or exp glucagon like peptide receptor agonist/ or exp glucagon like peptide 1/ or exp dipeptidyl peptidase IV inhibitor/ or exp dipeptidyl peptidase IV/ or incretin.mp.

3 (glucagon like peptide 1 or dipeptidyl peptidase).ti,ab.

4 exp perioperative period/ or (perioperat or peri-operat* or surgery).ti,ab.*

5 (intensive care or critical care).mp,ti,ab. or exp intensive care/

6 2 or 3

7 4 or 5

8 6 and 7

9 1 and 8

3.2. Filters:

None

3.3. Date Run:

13-2-2018

3.4. Results:

786

3.5. Actions:

Exported as File, Format: .ris

Saved: Search Embase SR GLP1 OK IC 5-3-2017.ris

Added to Endnote: Import file, select file, option: Cochrane (Wiley)

4. First deletion of Duplicates

After entrance of 3 databases: 135 + 269 + 786 = 1190

64 duplicates deleted, 1126 results left.

5. Import to Rayyan

Export Endnote file via export as txt file with endnote output style.

Saved as: EndnoteLibPubCochEmb13-2-2017.txt

Imported in Rayyan

6. Screening of articles

(Title and abstract screening for all articles that were **clinical trials** on **ICU** or **OR** patient populations with either a **DPP-IV inhibitor** or **GLP-1 RA**)

Independent screening by 2 authors. 3rd author for decision on all conflicts.

Result: 1047 Articles screened on title and abstract. 61 included, 986 excluded.)

Entered in new database Endnote: SR GLP1 full articles selection

7. First deletion of Duplicates

Abstract deleted if final study article was published and included in database.

29 records deleted 33 left for full text review.

8. Full text review

3 authors contacted, retrieved 2 original articles, 2 corresponding abstracts were excluded.

PRISMA 2009 Flow Diagram for Study

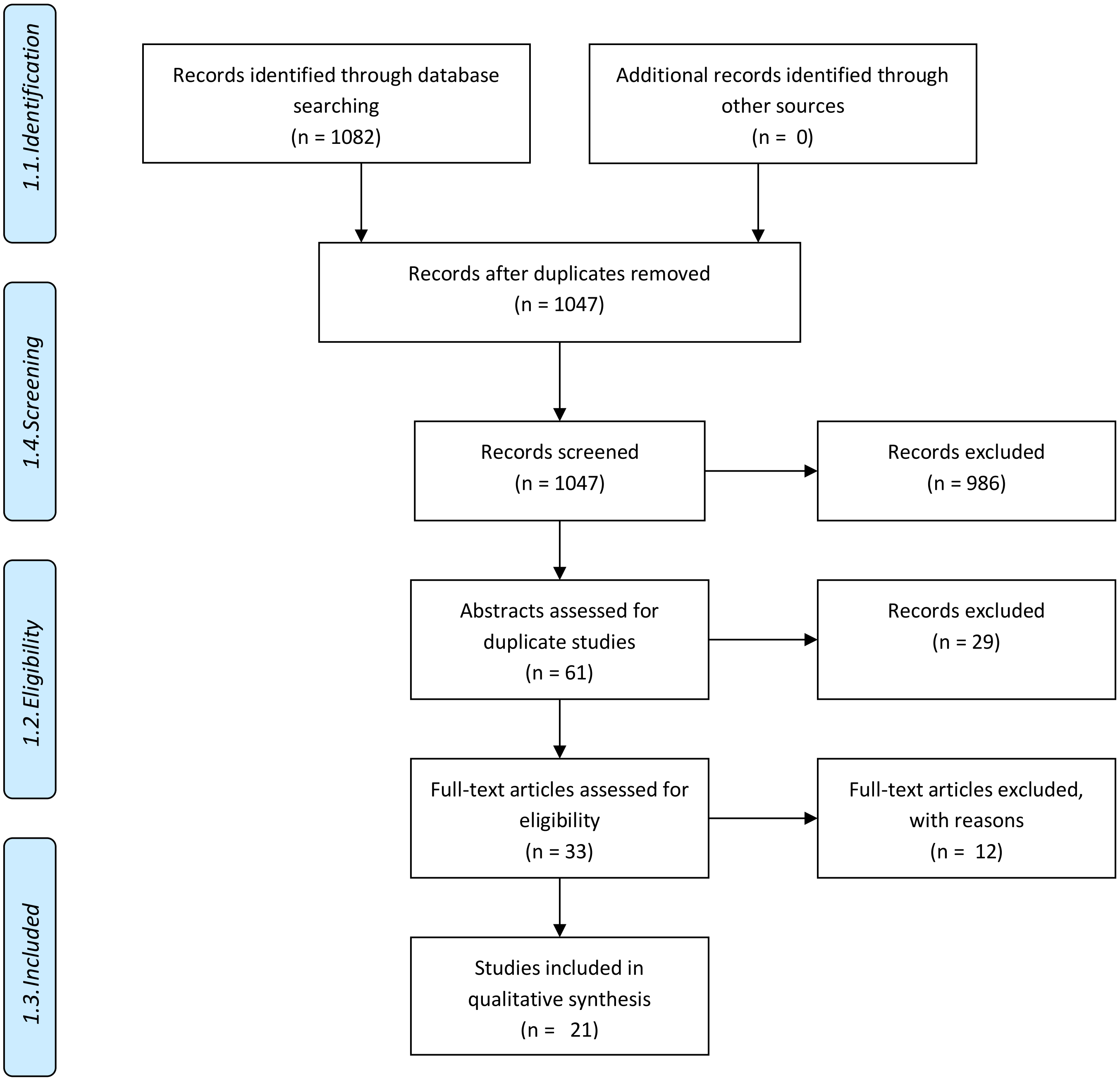

Supplement: Supplementary file 1 — Methodology of systematic review on incretins in peri-operative and intensive care (PDF 88 kb) [file 13054_2018_2197_MOESM1_ESM.pdf]
